# Detection of autoantibodies against reactive oxygen species modified glutamic acid decarboxylase-65 in type 1 diabetes associated complications

**DOI:** 10.1186/1471-2172-12-19

**Published:** 2011-03-08

**Authors:** Mohd Wajid Ali Khan, Kamalpreet Banga, Subhash N Mashal, Wahid Ali Khan

**Affiliations:** 1 Department of Biochemistry, Faculty of Medicine and Health Sciences - Gomail, University of Aljabal Algharbil, Zawia-16418, Libya; 2 Department of Biochemistry, Faculty of Medicine, J. N. Medical College, Aligarh Muslim University, Aligarh-202002, India; 3 Department of Community Medicine, Faculty of Medicine and Health Sciences - Gomail, University of Aljabal Algharbil, Zawia-16418, Libya; 4 Department of Pathology, Faculty of Medicine and Health Sciences - Gomail, University of Aljabal Algharbil, Zawia-16418, Libya; 5 Department of Clinical Biochemistry, College of Medicine and Medical Science, King Khalid University, Abha-61421, Kingdom of Saudi Arabia

## Abstract

**Background:**

Autoantibodies against glutamate decarboxylase-65 (GAD_65_Abs) are thought to be a major immunological tool involved in pathogenic autoimmunity development in various diseases. GAD_65_Abs are a sensitive and specific marker for type 1 diabetes (T1D). These autoantibodies can also be found in 6-10% of patients classified with type 2 diabetes (T2D), as well as in 1-2% of the healthy population. The latter individuals are at low risk of developing T1D because the prevalence rate of GAD_65_Abs is only about 0.3%. It has, therefore, been suggested that the antibody binding to GAD_65 _in these three different GAD_65_Ab-positive phenotypes differ with respect to epitope specificity. The specificity of reactive oxygen species modified GAD_65 _(ROS-GAD_65_) is already well established in the T1D. However, its association in secondary complications of T1D has not yet been ascertained. Hence this study focuses on identification of autoantibodies against ROS-GAD_65 _(ROS-GAD_65_Abs) and quantitative assays in T1D associated complications.

**Results:**

From the cohort of samples, serum autoantibodies from T1D retinopathic and nephropathic patients showed high recognition of ROS-GAD_65 _as compared to native GAD_65 _(N-GAD_65_). Uncomplicated T1D subjects also exhibited reactivity towards ROS-GAD_65_. However, this was found to be less as compared to the binding recorded from complicated subjects. These results were further proven by competitive ELISA estimations. The apparent association constants (AAC) indicate greater affinity of IgG from retinopathic T1D patients (1.90 × 10^-6 ^M) followed by nephropathic (1.81 × 10^-6 ^M) and uncomplicated (3.11 × 10^-7 ^M) T1D patients for ROS-GAD_65 _compared to N-GAD_65_.

**Conclusion:**

Increased oxidative stress and blood glucose levels with extended duration of disease in complicated T1D could be responsible for the gradual formation and/or exposing cryptic epitopes on GAD_65 _that induce increased production of ROS-GAD_65_Abs. Hence regulation of ROS-GAD_65_Abs could offer novel tools for analysing and possibly treating T1D complications.

## Background

In autoimmune diabetes the autoantibodies have always been important for clinical interest due to their potential role in screening, diagnosis, monitoring treatment of effectiveness and prognosis. The GAD_65_Abs are often considered to be an epiphenomenon resulting from the autoimmune destruction of the pancreatic beta cells in T1D. Previous studies suggest that they are involved in antigen processing and presentation and thus modulate the immune response [1]. Because of the high diagnostic sensitivity for autoimmune diabetes, the presence of GAD_65_Ab is currently used to identify subjects at high risk for the disease [2]. GAD_65_Abs are detected in about 60% of new-onset cases of type 1 diabetes [3], and high levels of these autoantibodies were also reported in diabetic patients with secondary complications (such as retinopathy and nephropathy), thus leading cause of blindness and renal failure [4,5]. The exact etiology behind these complications is not completely clear.

In our recent study; ROS modified GAD_65 _was found to be more immunogenic in T1D than its native form [6]. GAD_65_Abs in T1D are predominantly directed at conformational epitopes located in the middle region of the molecule, whereas they also recognize linear epitopes and epitopes located in the middle, COOH- and NH_2_-terminuses [7,8]. Shifts in GAD_65 _epitopes were detected in a subgroup of newly diagnosed children within the first 12 months after disease onset [9]. Moreover, epitope spreading has gained credence as a major driver underlying autoimmunity [10].

Growing evidence suggests that ROS plays an important role in the initiation and progression of diabetes and its associated complications [11]. These increased levels of free radicals pose a direct toxic effect on GAD_65 _and increase its immunogenicity [6]. Specificity of autoantibodies for epitopes on GAD_65 _and their levels may be a better indicator of impending or actual destruction of islet β-cells and increasing complications associated with diabetes.

In the view of the above mentioned studies we hypothesized some possible link between diabetic associated complications and presence of ROS-GAD_65_Abs. To prove this, binding characteristics of serum autoantibodies from uncomplicated and complicated (nephropathic and retinopathic) T1D patients were assessed with N-GAD_65 _and ROS-GAD_65 _by direct binding and competitive ELISA. The avidity of modified GAD_65 _was also evaluated by precipitate titration curve in different diabetic groups.

## Results

### ROS modification of GAD_65_

ROS directed modification of GAD_65 _studied previously by our group showed marked structural changes [6]. Khan *et al*., demonstrated that hyperchromicity and tryptophan specific fluorescence for modified GAD_65 _was found to be significantly higher than native GAD_65 _and the spectral analysis also showed blue shift of 10 nm in modified GAD_65 _over native GAD_65_. Far-UV-CD spectropolarimetry of ROS-GAD65 exhibited significant changes in secondary structural elements compared to its unmodified form decrease in α-helix and an increase of in β-sheet, random coil and turns was observed [6].

### Detection of autoantibodies against N-GAD_65 _and ROS-GAD_65_

**Figure 1:**
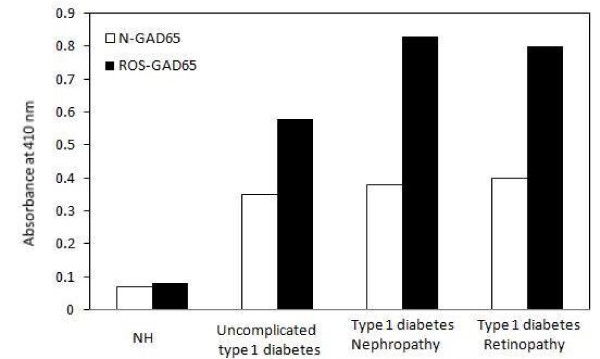
**Detection of serum autoantibodies against both native and modified GAD**_**65 **_**in all subjects**. Direct binding ELISA of 1:100 diluted uncomplicated and complicated (nephropathy and retinopathy) T1D serum samples. Control samples were served as controls. Serum autoantibodies reactivity of all four groups was checked towards N-GAD_65 _(□) and ROS-GAD_65 _(■). Microtitre plates were individually coated with N-GAD_65 _and ROS-GAD_65 _(20 μg/ml). Each histogram represents the mean ± SD values.

In a pilot study serum samples from uncomplicated and complicated T1D patients were screened for autoantibodies against N-GAD_65 _(GAD_65_Abs) and ROS-GAD_65 _(ROS-GAD_65_Abs) using unmodified and ROS-modified GAD_65 _as antigens. From Figure 1, we observed that sera from normal human (NH) subjects showed very low level of reactivity towards N-GAD_65 _[optical density (OD); 0.07 ± 0.02] or ROS-GAD_65 _(OD; 0.08 ± 0.02). Conversely, serum autoantibodies of uncomplicated T1D patients showed significant binding with ROS-GAD_65 _(OD; 0.58 ± 0.04, *p < .0001*) as compared to N-GAD_65 _(OD; 0.35 ± 0.03). Moreover, sera from diabetic nephropathic (OD; 0.83 ± 0.03, *p < .0001*) and diabetic retinopathic (OD; 0.80 ± 0.05, *p < .0001*) patients exhibited statistically higher significant differences in the binding pattern of serum autoantibodies with ROS-GAD_65 _as compared to N-GAD_65 _(nephropathic; 0.38 ± 0.05 and retinopathic; 0.40 ± 0.04).

OD was considered as a direct measure of the concentrations of autoantibodies in the serum samples. In all the study groups there were higher levels of reactivity of modified antigen as compared to its native form. NH samples showed 14% increased and uncomplicated serum samples exhibited 65.7% increased reactivity towards ROS-GAD_65 _as compared to N-GAD_65_. Whereas, nephropathic and retinopathic T1D complicated subjects showed an increase of 118.4% and 100% respectively, in the reactivity with modified antigen when compared with unmodified antigen.

The binding specificities of serum autoantibodies from uncomplicated and complicated (Nephropathic and retinopathic) T1D patients were evaluated by competitive ELISA using N-GAD_65 _and ROS-GAD_65 _as inhibitors. Significantly higher recognition of modified antigen was observed by the serum autoantibodies from nephropathic [70.3 ± 8.2 mean maximum percent inhibition (MMPI)] and retinopathic patients (74.5 ± 6.5 MMPI] as compared to uncomplicated T1D serum samples (50.6 ± 7.2 MMPI). N-GAD_65 _exhibited no marked difference in recognition of serum IgG from both complicated [nephropathy (39.2 ± 5.4 MMPI) and retinopathy (41.1 ± 5.3 MMPI)] and uncomplicated (35.2 ± 5.9 MMPI) subjects of T1D, Table 1. Normal humans showed very less or negligible percent inhibition with either of the antigens [N-GAD_65 _(7.3 ± 3.6 MMPI) and M-GAD_65 _(7.2 ± 3.2 MMPI)].

**Table 1:** Inhibition of serum IgG from uncomplicated T1D, complicated T1D and control subjects towards native and modified GAD_65_.

Maximum percent inhibition at 20 μg/ml								
Serum	Uncomplicated T1D		Nephropathic T1D		Retinopathic T1D		Controls	
	**N-GAD**_65_	**M-GAD**_65_	**N-GAD**_65_	**M-GAD**_65_	**N-GAD**_65_	**M-GAD**_65_	**N-GAD**_65_	**M-GAD**_65_
1	36	54	40	78	39	85	-	-
2	38	40	42	66	37	79	-	7
3	47	61	54	79	44	57	7	11
4	37	55	41	70	41	68	-	-
5	43	49	48	66	32	65	-	-
6	35	54	35	74	37	79	-	10
7	40	60	44	56	43	76	10	7
8	26	68	39	73	51	57	-	-
9	22	37	30	59	36	70	7	9
10	38	56	37	62	50	77	8	5
11	45	63	42	57	40	70	-	7
12	34	50	29	59	48	69	-	-
13	46	55	37	76	50	79	5	9
14	41	52	36	69	36	75	-	-
15	33	39	39	78	33	81	-	-
16	21	48	28	79	30	83	6	7
17	19	41	26	69	28	78	8	6
18	26	37	27	75	37	79	6	7
19	31	45	35	81	35	82	9	8
20	28	47	39	80	40	80	-	-

Mean ± SD	35.2 ± 5.9	50.6 ± 7.2	39.2 ± 5.4	70.3 ± 8.2*	41.1 ± 5.3	74.5 ± 6.5*	7.3 ± 3.6	7.2 ± 3.7

N-GAD_65 _and M-GAD_65 _represents Native GAD_65 _and modified GAD_65_.

NH represents normal humans as control.

The ELISA plates were coated with N-GAD_65 _and M-GAD_65 _(20 μg/ml).

ROS-GAD_65 _and N-GAD_65 _were used as inhibitor.

* *p < 0.001 *vs ROS-GAD_65_Abs in uncomplicated T1D.

### Estimation of protein bound carbonyl compounds in serum samples

In vivo carbonyl content was considered a biomarker of oxidative stress. Oxidative stress levels were estimated for every patient of each group of T1D (Table 2). Data showed significant increase in serum protein bound carbonyl contents (*p < 0.001*) in complicated subjects as compared to uncomplicated T1D patients. Complicated subjects, T1D who had retinopathy (3.9 ± 0.31 nmoles/mg protein) exhibited higher amounts of protein bound carbonyl content as compared to nephropathic (3.4 ± 0.28 nmoles/mg protein) T1D patients.

**Table 2:** Clinical and laboratory data from complicated and uncomplicated T1D patients; normal human subjects serve as controls.

Subjects	Number of sera	Age (years ± SD)	Gender (M:F)	Smoking duration n(years ± SD)	Duration of disease (years ± SD)	Fasting blood glucose (mg/dl)	HbA_1C _(%)	Hyperten-sion 140/90 (%)	Carbonyl Content (nmol/mg protein)
Uncomplic-ated T1D	60	30 **± **09	37:23	8(5 ± 3.4)	09 ± 5.6	238 ± 27^#^	7.9 ± 0.7	36(60)	3.0 **± **0.22^#^
Complicated T1D Nephropathy	20	37 ± 11	12:8	14(6 ± 3.8)	14 ± 4.9	311 ± 21*	8.8 ± 0.6*	17(85)	3.4 **± **0.28*
Complicated T1D Retinopathy	20	42 ± 14	11:9	17(8 **± **3.6)	17 ± 4.3	335 **± **17*	9.3 **± **0.7*	16(80)	3.9 **± **0.31*
Control	50	32 ± 8	28:22	--	--	96 ± 11.2	5.8 ± 0.4	4(8)	2.1 **± **0.17

Data are means **± **SD or n represents number of smokers from given total respective subjects. For blood glucose estimations, blood was collected in oxalated fluoride containers and the assays were performed immediately. Hypertension is defined as sitting systolic blood pressure ≥ 140 mmHg and/or diastolic blood pressure ≥ 90 mmHg or the use of antihypertensive medication. Signs * and ^# ^represents *p *values < 0.001 and < 0.05 respectively.

### Quantification of apparent association constant

**Figure 2:**
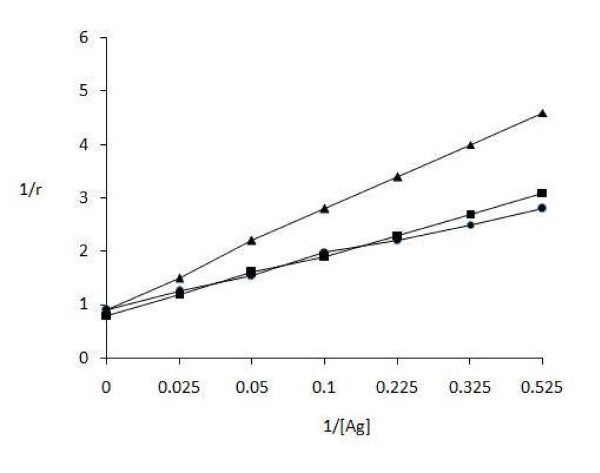
**Determination of apparent association constants for ROS-GAD**_**65 **_**by Langmuir plot**. Langmuir plot of reciprocal of bound antigen concentration to antibody (1/r) versus reciprocal of free antigen concentration (1/[Ag]). Antigen and antibody binding between ROS-GAD_65 _and IgG isolated from nephropathic (-■-) serum no.3, retinopathic (-●-) serum no. 6 of complicated T1D and uncomplicated (-▲-) (serum no. 11) T1D subjects. Each value represents mean ± SD of four independent assays.

**Figure 3:**
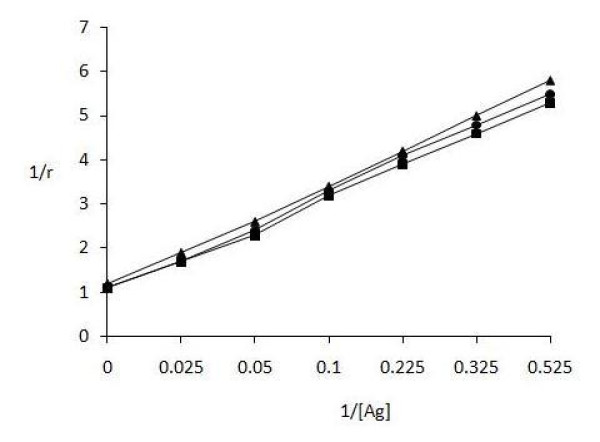
**Determination of apparent association constant for N-GAD**_**65 **_**by Langmuir plot**. Langmuir plot of reciprocal of bound antigen concentration to antibody (1/r) versus reciprocal of free antigen concentration (1/[Ag]). Antigen and antibody binding between N-GAD_65 _and IgG from nephropathic (-■-) serum no.3, retinopathic (-●-) serum no. 6 complicated T1D and uncomplicated (-▲-) (serum no. 11) T1D subjects. Each value represents mean ± SD of four independent assays.

The amount of antigen bound to antibody was also evaluated by quantitative precipitin titration curve. IgG of uncomplicated (serum no. 11) and complicated [nephropathic (serum no. 3) and retinopathic (serum no. 6)] subjects was purified by affinity chromatography on Protein A-Agarose column. The purified IgGs were found to elute in a single symmetrical peak. Varying amounts of modified GAD_65 _(0-40 μg) were mixed with 100 μg of patient's IgG and incubated for 2 h at 37°C and overnight at 4°C. Microsurface adsorption-spectral correction (MSASC) technique showed the interaction of IgG with modified protein. Langmuir equation was used to estimate AAC of complicated and uncomplicated T1D samples for ROS-GAD_65 _(Figure 2) and was computed to be 1.81 × 10^-6 ^M and 1.90 × 10^-6 ^M for T1D nephropathic and retinopathic patients respectively. Uncomplicated T1D showed 3.11 × 10^-7 ^M AAC. A maximum of 23 μg and 20 μg of ROS-GAD_65 _was bound to 84 μg and 87 μg of IgG from T1D nephropathic and retinopathic subjects respectively. However uncomplicated T1D patients exhibited 28 μg of IgG bound with 76 μg of ROS-GAD_65_. No appreciable differences were observed in the AACs calculated for N-GAD_65 _in same above mentioned serum samples of complicated (nephropathic; 2.87 × 10^-7 ^M and retinopathic; 2.73 × 10^-7 ^M) and uncomplicated T1D patients (2.63 × 10^-7 ^M) as shown in Figure 3.

## Discussion

The levels of ROS increase many folds during T1D via formation of sorbitol by polyol pathways, regeneration of cofactors NADPH and NAD^+ ^by NAD(P)H oxidase and glycation reactions [12,13]. Long term association of proteins with high concentrations of hydrogen peroxide and free radical intermediates results in protein modification both at the amino acid and protein levels [14]. This statement is supported by our previous findings based on the structural characterization of native and ROS-GAD_65 _(increased UV-absorbance and tryptophan fluorescence and changes in secondary structural elements) [6].

Significant recognition of ROS-GAD_65 _by serum autoantibodies of complicated T1D patients was estimated when compared with autoantibodies from uncomplicated T1D subjects. N-GAD_65 _did not show any marked differences in recognitions of circulatory autoantibodies from complicated and uncomplicated T1D subjects. The affinity of GAD_65_Abs was found to be higher in subjects who had developed T1D with neuropathy [15] or autoimmune polyendocrine disorders [16]. In control normal humans, negligible binding was observed with either of the antigens.

From the cohort, twenty serum samples each from complicated (nephropathic and retinopathic) and uncomplicated T1D patients were selected and binding affinities of circulating autoantibodies with N-GAD_65 _and ROS-GAD_65 _were ascertained by competitive ELISA. Twenty serum samples from normal humans were taken under the same experimental condition as controls. A characteristic difference was observed in the pattern of inhibition ELISA assays obtained from complicated and uncomplicated T1D subjects in respect to ROS-GAD_65 _antigen. This indicates that the ROS-modified GAD_65 _is an effective inhibitor showing substantial higher titres of circulating autoantibodies in complicated T1D subjects as compared to uncomplicated T1D. Moreover; amongst the complicated patients, retinopathic showed highest recognition for ROS GAD_65 _as compared to nephropathic and uncomplicated patients. Further the study also elucidates that with increased duration of disease and poor glycemic control leads to increased oxidative stress and hence the complications. The oxidative stress was further ascertained by the levels of protein bound carbonyl content in patients which is a biomarker of protein oxidation [17]. Type 1 diabetes retinopathic patients had highest carbonyl content followed by nephropathic and uncomplicated respectively. Possible this heightened state of oxidative stress leads to extensive in vivo GAD65 antigen modifications. Thus GAD_65 _of complicated T1D patients presents more number of epitopes that resemble in vitro ROS modified GAD_65 _and conceivably generates significant number of autoantibodies. GAD_65_Ab titers are higher and more prevalent in patients with other associated autoimmune diseases such as thyroiditis [18]. The strong dependence of conformation of protein for autoantibody recognition, blocking experiments [19] and recombinant Fab using monoclonal antibodies [16,20] has been useful for determining conformational GAD_65_Ab epitopes.

During immune-pathophysiology significant amounts of circulating immune complexes are formed and deposited in kidneys leading to diabetic nephropathy, retinopathy and other tissues causing severe injury [21]. As we discussed in this study continuous long durations of increased levels of ROS cause increase in antigenic determinants on GAD_65_. So, the avidity of GAD_65 _became more complex and gain increased strength of binding because of interdependency of epitopes. Figure 2 and 3 clearly indicate better recognition of ROS-GAD_65 _than N-GAD_65 _by IgG isolated from retinopathic T1D subjects followed by nephropathic and uncomplicated T1D patient. The enhanced recognition of ROS-GAD_65 _by retinopathic T1D patient IgG showed the possible participation of oxidative stress and long duration of disease as given in Table 2 that might have role in in vivo modification of GAD_65 _inducing the molecule to express its cryptic epitopes.

## Conclusion

In conclusion, significantly high levels of circulating ROS-GAD_65_Abs were detected in complicated (retinopathic and nephropathic) as compared to uncomplicated T1D patients. This risk of the disease may be exemplified due to acceleration in the formation of free radicals with gradual increase in duration of disease. This leads to conformational alterations in N-GAD_65 _protein which could increase or expose cryptic epitopes. Dynamic changes in the GAD_65_Abs binding pattern suggest subsequent epitopes spreading with disease progression. This could be one of the etiologies of increased GAD_65_Ab immunogenicity that implicated in T1D complications. Measurement of these autoantibodies could be shown to be useful in assisting the prediction for the development of T1D progression/or complications. Reduction in the levels of ROS may lead to decrease in in vivo GAD_65 _molecules modification thus, leads to delay in the progression of complications. Hence antioxidants may play important role in the treatment.

## Methods

### Human serum samples

In the present study 100 T1D (60 uncomplicated and 40 complicated) and 50 control normal human (NH) subjects were investigated. All the patients were on the insulin treatment with suitable doses depending on the clinical examinations. All the serum samples of patients were collected from the laboratory of Endocrinology, Department of Medicine (J. N. Medical College and Hospital, A. M. University, Aligarh, India) and their clinical features are shown in Table 2. Approximately 20 ml of fasting venous blood was collected from each subject. For estimation of glucose, blood was taken in oxalated fluoride containers and the assays were performed immediately. Isolated serum samples from all subjects were heated at 56°C for 30 min to deactivate complement protein and stored at -20°C with sodium azide. The categories for diabetic complications were mutually exclusive. Normal humans served as controls, age and sex matched with no family history of diabetes. All groups underwent periodic examinations. All subjects gave informed consent to the analysis and the study had Ethics Committee approval. Patient classification is summarized as follows.

#### Uncomplicated patients

These patients (n = 60) have had T1D and remained free from any complications (retinopathy and nephropathy). These patients are negative proteinuria.

#### Nephropathic patients

These patients (n = 20) had T1D and all were proteinuria positive (urinary protein excretion rates ≥300 mg/24 h) in the absence of hematuria or infection in midstream urine samples.

#### Retinopathic patients

These patients (n = 20) had retinopathy defined as having more than five dots or blots per eye; hard or soft exudates and vitreous hemorrhage.

### Preparation of Antigen

Human-GAD_65 _(G-2126, Type II: from E. coli, Sigma, St. Louis, MO, USA) was modified with hydroxyl radicals. Briefly, solution (3.0 ml total volume) of N-GAD_65 _(100 mg/ml) in 50 mM sodium phosphate buffer, pH 7.4, was irradiated with 254 nm UV light for 30 min at room temperature in the presence of 10 mM hydrogen peroxide (Genei, Bangalore, India). After modification, extensive dialysis has been was done with 50 mM sodium phosphate buffer to remove excess hydrogen peroxide and hydroxyl radicals. Protein concentration determined by Bradford's method [22].

### Protein bound carbonyl groups

Protein bound carbonyl groups from sera of different diabetic groups and NH subjects were analyzed according to Levine *et al. *[23] and the results were expressed as the number of nanomoles of carbonyl per mg of sample protein using a ε_379 _= 22,000 M^-1^.cm^-1^. Protein concentration of the samples was determined by Bradford's method [22].

### Elisa

Direct binding ELISA was performed on polystyrene microtitre flat bottom plates (NUNC, Roskilde, Denmark), as described previously [6,24]. Briefly plates were coated with 100 μl of respective antigen (20 μg/ml) for 2 h at room temperature and overnight at 4°C. The plates were washed with TBS-T (20 mM Tris, 2.68 mM KCl, 150 mM NaCl, pH 7.4, containing 0.05% Tween-20) and unoccupied sites were blocked with 150 μl of 1.5% BSA in TBS (10 mM Tris, 150 mM NaCl, pH 7.4) for 4-6 h at room temperature. The test serum (diluted 1:100) in TBST (100 μl per well) was adsorbed for 2 h at room temperature and overnight at 4°C. Bound antibodies were assayed with anti-human IgG alkaline phosphatase conjugate (Sigma, St. Louis, MO, USA) using para-nitrophenyl phosphate (Sigma, St. Louis, MO, USA) as substrate. The absorbance of each well was monitored at 410 nm on an automatic microplate reader (Labsystem Multiskan EX, Helsinki, Finland).

### Competitive ELISA

The antigenic specificity of modified GAD_65 _was determined by competitive ELISA [6,24]. Varying concentrations of inhibitors (0-20 μg/ml) were allowed to interact with a constant amount of serum antibody (1:20 diluted serum) for 2 h at room temperature and overnight at 4°C. The immune complex thus formed was incubated in the wells and the bound antibody levels were detected as in direct binding ELISA.

The percent inhibition was calculated using the formula:

Where A _inhibited _is the absorbance at 20 μg/ml of inhibitor concentration and A _uninhibited _the absorbance at zero inhibitor concentration.

### IgG isolation

Immunoglobulin G was isolated from uncomplicated and complicated T1D sera on Protein A-Sepharose CL-4B column (Genei, Bangalore, India) [25]. The homogeneity of isolated IgG was checked by 7.5% polyacrylamide gel electrophoresis.

### Quantitation of antigen-antibody immune complex

One hundred micrograms of IgG was incubated with varying amounts of ROS-GAD_65 _antigen in an assay volume of 500 μl. The mixture was incubated for 2 h at room temperature and overnight at 4°C. The immune complexes were pelleted, washed twice with PBS and dissolved in 250 μl of 1 N NaCl. Protein concentrations were measured by colorimetric method [22]. The binding data were analyzed for antibody affinity [26].

### Statistical evaluation

The values are given as arithmetic mean ± SD wherever indicated. Multiple comparisons were analyzed by student t test using SPSS16 software program and p < 0.05 was considered to be statistically significant.

## Authors' contributions

MWAK designed and carried out all of the experiments and analysed the results. KB participated in data analyses. SNM helped in drafting the manuscript. WAK helped to carry out some ELISA experiments and in writing the discussion. All authors read and approved the final manuscript.
